# Survival Implications Associated with Variation in Mastectomy Rates for Early-Staged Breast Cancer

**DOI:** 10.1155/2012/127854

**Published:** 2012-08-08

**Authors:** John M. Brooks, Elizabeth A. Chrischilles, Mary Beth Landrum, Kara B. Wright, Gang Fang, Eric P. Winer, Nancy L. Keating

**Affiliations:** ^1^College of Pharmacy, University of Iowa, Iowa City, IA 52242, USA; ^2^College of Public Health, University of Iowa, Iowa City, IA 52242, USA; ^3^Department of Health Care Policy, Harvard Medical School, Boston, MA 02115, USA; ^4^Eshelman School of Pharmacy, University of North Carolina, Chapell Hill, NC 27599, USA; ^5^Department of Medical Oncology, Dana Farber Cancer Institute, Boston, MA 02115, USA; ^6^Division of General Internal Medicine, Harvard Medical School, Brigham and Women's Hospital, Boston, MA 02120, USA

## Abstract

Despite a 20-year-old guideline from the National Institutes of Health (NIH) Consensus Development Conference recommending breast conserving surgery with radiation (BCSR) over mastectomy for woman with early-stage breast cancer (ESBC) because it preserves the breast, recent evidence shows mastectomy rates increasing and higher-staged ESBC patients are more likely to receive mastectomy. These observations suggest that some patients and their providers believe that mastectomy has advantages over BCSR and these advantages increase with stage. These beliefs may persist because the randomized controlled trials (RCTs) that served as the basis for the NIH guideline were populated mainly with lower-staged patients. Our objective is to assess the survival implications associated with mastectomy choice by patient alignment with the RCT populations. We used instrumental variable methods to estimate the relationship between surgery choice and survival for ESBC patients based on variation in local area surgery styles. We find results consistent with the RCTs for patients closely aligned to the RCT populations. However, for patients unlike those in the RCTs, our results suggest that higher mastectomy rates are associated with *reduced* survival. We are careful to interpret our estimates in terms of limitations of our estimation approach.

## 1. Introduction

Despite randomized controlled trial (RCT) evidence suggesting survival equivalence between breast conserving surgery with radiation (BCSR) and mastectomy for woman with early-stage breast cancer (ESBC) [1–5] and a nearly 20-year-old guideline from the National Institutes of Health Consensus Development Conference recommending BCSR over mastectomy because it preserves the breast [6], mastectomy remains widely used [7, 8]. In fact, recent evidence suggests that mastectomy rates are increasing [7, 9]. In addition, higher-staged patients have been more likely to receive mastectomy [8, 10]. These observations suggest that some patients and their providers believe that mastectomy has advantages over BCSR and these advantages increase with stage. These beliefs may persist because the randomized controlled trials (RCTs) that served as the basis for the NIH guideline were populated mainly with lower-staged, younger patients with few comorbidities [1–5].

In an analysis of the Surveillance, Epidemiology and End Results (SEER)-Medicare observational database [11] for ESBC patients using risk-adjustment methods, Keating et al. found no difference in mortality risk for mastectomy relative to BCSR for patients similar to those RCT samples (low-risk tumors—stage I, little comorbid illness, and age ≤ 70) but higher average mortality risk across all ESBC patients [8]. These results imply a survival disadvantage for mastectomy in patients with larger tumors, more comorbid illnesses, or advanced age. In another risk-adjustment study using SEER-Medicare data, Schonberg et al. found that mastectomy had a survival disadvantage relative to BCSR that increased with stage [10]. The risk-adjustment estimators used in both of these analyses only adjusted for measured covariates. For the estimates from these analyses to be unbiased it must be assumed that unmeasured covariates affecting surgery choice are unrelated to survival [12, 13]. Keating et al. addressed this issue by assessing whether their results were robust to plausible imbalances in unmeasured covariates across surgery choices. This analysis found, at the very least, no evidence of a survival advantage associated with mastectomy. 

Instrument variable (IV) estimators also are available to estimate treatment effectiveness using observational data. IV estimators are distinct from risk-adjustment estimators in both the assumptions required to yield unbiased estimates and in the interpretation of the eventual estimates [14–18]. Risk-adjustment estimators assume that unmeasured covariates affecting treatment choice are unrelated to outcomes and yield parameters that are properly interpreted as estimates of the average treatment effectfor the patients that received treatment [12–16, 19, 20]. In contrast, IV estimators yield estimates of a local average treatment effect, that is, the average effect for the subset of patients whose treatment choices were affected by measured factors called “instruments” [17, 21, 22]. IV estimators require instruments with strong relationships with treatment choice [23]. IV estimators work under the assumption that the instruments used in the analysis have no direct effect on outcome and are independent of unmeasured covariates that affect outcome. Based on this assumption, IV estimators have been likened to natural experiments in treatment choice [24]. Previous IV studies assessed the effect of surgery choice for ESBC patients but were limited geographically, had small sample sizes, and used instruments with weak relationships with surgery choice which resulted in imprecise parameter estimates [15, 25, 26]. These pitfalls are avoided in this study by using the SEER-Medicare database that included ESBC patients from across the United States and by using an instrument that strongly predicts surgery choice. Our instrument exploits variation in local area physician practice styles that has been shown to be a practical and rich source for instrument development [15, 25, 27–31]. This approach conjectures that patients residing in areas with physicians that have preferences for a particular treatment are more apt to receive that treatment and that unmeasured confounding variables are unrelated to this access. Our algorithm to measure local area practice styles has been shown to explain a larger portion of treatment variation than other approaches and it effectively balances measured confounding variables [31, 32]. Mastectomy rates vary significantly across the United States [8], suggesting that local area practice styles for ESBC patients vary regionally. From 1992–2002, mastectomy rates for ESBC patients were 40.4% in Connecticut, 59.2 in Utah, and 67.4% in Iowa [8].

## 2. Materials and Methods

### 2.1. Data and Sample

We used the 1992–2002 SEER-Medicare databases and employed the same base inclusion/exclusion criteria used in the Keating study [8, 11]. We identified 69,140 patients with stage I or II breast cancer that were enrolled in parts A and B of fee-for-service Medicare from 12 months before diagnosis through 9 months after diagnosis and had primary surgery (either breast conserving surgery or mastectomy). To compute local area mastectomy practice styles, we further excluded 1,005 patients that did not have a valid residence ZIP code, whose residence ZIP code was outside of a SEER area, or lived in an isolated island location within the Hawaii SEER registry. The remaining 68,135 patients were used to estimate local area mastectomy practice styles. Our dependent variable was 7-year survival after diagnosis, and our focus was comparing patients receiving mastectomy alone to those receiving BCSR. To estimate the effects of surgery choice on survival we further excluded patients with less than 7 years of followup (*N* = 32,683) and patients that either received BCS without radiation or mastectomy with radiation (*N* = 6,777) leaving a final estimation sample of 28,675 patients. Models were also estimated using 6-year and 8-year survival as robustness checks for our estimates.

### 2.2. Instrumental Variable Approach

We estimated local area mastectomy practice styles using the driving area for clinical care (DACC) method [31, 32]. All ESBC patients with valid ZIP codes living in SEER areas (*N* = 68,135) were used. The DACC method produces ZIP-specific practice style measures that reflect the surgery choices for patients living within a driving distance of each ZIP code. For each patient residence ZIP code in the SEER-Medicare database, the DACC method expanded driving times around the ZIP code to add ESBC patients from additional ZIP codes until at least 50 patients were identified. For the set of patients associated with each ZIP code we then computed an area treatment ratio (ATR) as the ratio of the proportion of ESBC patients receiving mastectomy over the average predicted probability of the ESBC patients in the area to receive mastectomy. Predicted mastectomy probabilities for each patient were estimated using a multivariate logistic model of mastectomy choice over the 68,135 patients. The covariates used in this model are described in the variable specification section below. A ZIP code with an ATR greater than 1 suggests a local area practice style in which mastectomy is used at a rate higher than average given the characteristics of the ESBC patients in the area. An ATR less than 1 suggests a local area mastectomy practice style less than average. Our IV estimates rest on the assumption that the local area mastectomy practice style reflected by the ATR has no direct effect on survival other than through surgery choice and is unrelated to unmeasured covariates that affect survival. We assessed whether our results were robust to the number of ESBC patients used to define areas around ZIP codes by using 25 and 100-patient areas in separate analyses.

We applied the same two-stage least squares (2SLS) instrumental variable method used by the previous ESBC studies [15, 25, 26, 33] and the seminal IV study in health services research [34]. 2SLS yields consistent estimates regardless of the underlying error distributions in contrast to alternative estimators that rely on distributional assumptions which yield inconsistent results if the assumptions are wrong [35, 36]. In the first stage of the 2SLS we estimated a model of mastectomy choice across the 28,675 patients with 7 years of followup that included, among other covariates described below, the ATR-based instrument as an independent variable. The instrument was specified in this first stage model using a series of binary variables for each patient that placed the patient's residence ZIP code ATR within the distribution of ATRs across ZIP codes. For example, four binary variables were constructed to represent the five ZIP code groups defined by quintile cutoffs of the ATRs across ZIP codes. Patients were then assigned values for these binary variables based on the ATR of their residence ZIP code relative to the cutoffs (1 if patient residence ATR is within respective cut-off points, zero otherwise). The second stage of the 2SLS method was the 7-year survival model which includes all the non-instrument covariates from the first-stage model as independent variables plus the predicted mastectomy choice from the first stage. As a result of this 2-stage process, the parameter estimate associated predicted mastectomy choice in the second stage will be estimated using only the variation in mastectomy choice associated with the ATR instrument. Under the IV assumption, this estimated parameter provides a consistent estimate of the effect of mastectomy on survival.

To assess the robustness of our findings to alternative specifications, 2SLS models were run with the instrument specified using median, quintile, decile, and vigintile cutoffs of ATR values. 2SLS models were also estimated using the full sample and for patient subsets defined by stage, age, and the alignment of patients with clinical trial eligibility. In addition, we estimated a Hausman test statistic [37] for each 2SLS specification. 2SLS models in which the instrument is specified using more than two groups are also said to be overidentified and a Hausman test statistic can be estimated to test the null hypothesis of whether the patient groups identified using the instrument have no empirical relationship with survival beyond the effect of the instruments on surgery choice. A large value of the Hausman statistic rejects the null hypothesis. We also produced risk-adjustment estimates using linear probability models to provide a link between the risk-adjustment estimates from previous research and our IV estimates from the 2SLS estimator.

### 2.3. Variable Definitions

The multivariate logistic mastectomy choice model used as the basis for ATR estimation included patient covariates age at diagnosis, race, Hispanic ethnicity, marital status, diagnosis year, cancer history, tumor stage, tumor size, tumor grade, histology, estrogen and progesterone receptor status, comorbid illness, adjuvant chemotherapy use, and residence area average characteristics (education, income, and living in a metropolitan area). Characteristics of the hospital and physician were also specified. All covariates above were specified consistently with the Keating study and a full description of covariate measurement can be found there [8]. The dependent variable in 2SLS and linear probability models was a binary variable indicating 7-year survival after diagnosis (1 if patient survives 7 years, 0 otherwise). Surgery choice was defined using a binary variable (1 if patient received mastectomy, 0 if the patient received BCSR) based on accepted definitions using Medicare claims data [38–41]. In addition to the covariates above, the 2SLS and linear probability models also specified as covariates the proportion of ESBC patients that received breast conserving surgery alone and proportion of patients that received mastectomy plus radiation in the DACC-defined area around each patient residence ZIP code, and binary variables for each SEER area. These covariates were added to control for differences in the mix of ESBC patients not receiving mastectomy alone and BCSR across areas.

## 3. Results

Table 1 contains summary statistics for the estimation sample by surgery (BCSR versus mastectomy) choice and by the quintiles of the local area mastectomy practice style measure. Relative to patients choosing BCSR, mastectomy patients were generally older and sicker with higher-staged disease, larger tumors, a higher percentage of poorly differentiated tumors, and they had more comorbid conditions. In addition, patients receiving mastectomy were more apt to see physicians with lower surgical volume, be treated in smaller hospitals, live in nonmetro areas, and live in areas with lower area median per capita income. In contrast, there were few clear relationships between the patients grouped by the ATR measure of local area mastectomy practice style and measured covariates. From quintile 1 to quintile 5, the percentage of patients receiving mastectomy increases from 37.9% to 68.6%. While variation in the measured covariates is observed across the instrument-based quintile groups, these differences mostly do not trend with the mastectomy percentages across groups. Cochran-Armitage trend tests show statistically significant trends across the instrument groups only for moderate tumor grade, unknown tumor grade, hospital size, residence metro status, and area median income [42, 43]. 

**Figure 1:**
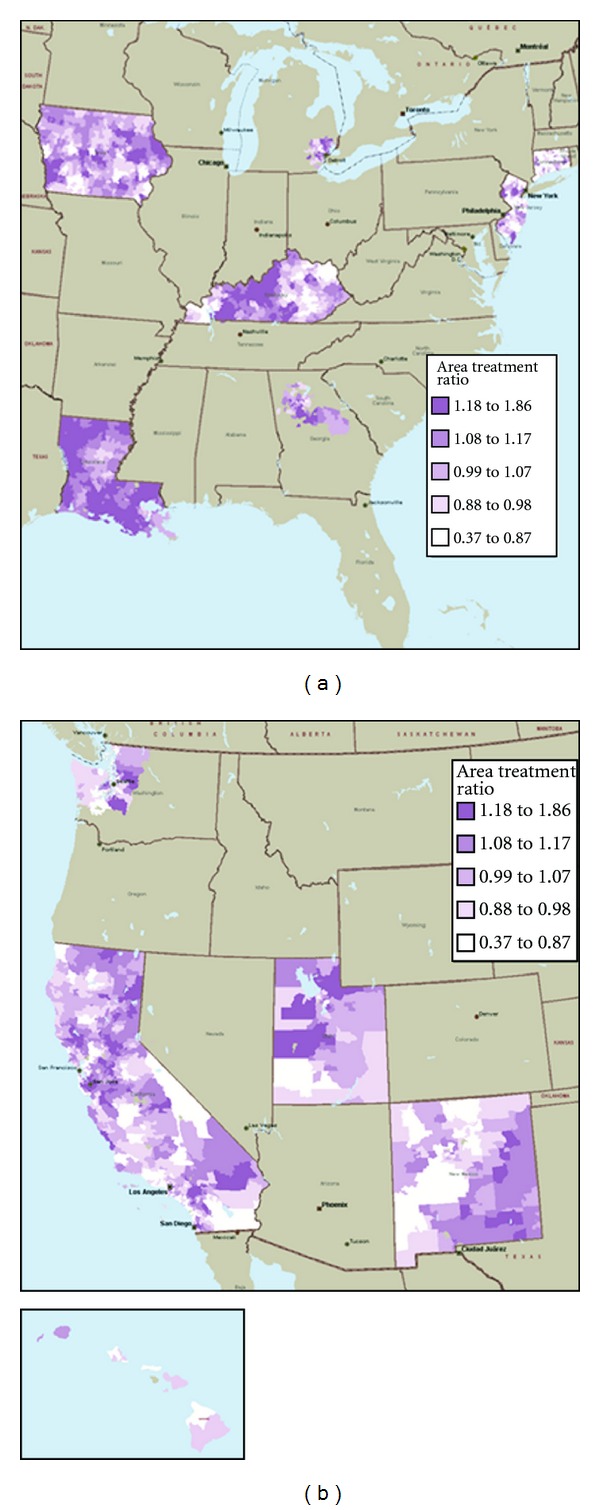
Zip-code level maps of mastectomy local area treatment ratios for SEER areas in the eastern United States.

Figures 1(a) and 1(b) contain maps showing mastectomy practice styles as measured by the area treatment ratios (ATRs) across SEER areas over the period 1992–2002. Clearly, substantial geographic variation in surgical choice for ESBC patients exists after controlling for differences in measured patient and provider characteristics. While some SEER-area specific variation is visible (e.g., Connecticut with few high mastectomy areas), both high and low mastectomy areas exist within each SEER area.

Table 2 contains estimates of the effect on 7-year survival of mastectomy relative to BCSR. The table contains both linear probability and IV estimates of the average 7-year survival effect of mastectomy relative to BCSR. As discussed above, these IV estimates are average mastectomy survival effects for those patients whose surgery choices would have differed had they lived in an area with a different local area mastectomy practice style. Table 2 contains IV estimates from the quintile instrument specification. Estimates from the other instrument specifications are comparable in magnitude and statistical significance and are available from the authors upon request. Our linear probability estimates for the full sample and for the patient subsets by trial status alignment are consistent with earlier risk-adjustment model findings. Mastectomy has a statistically significant negative impact on 7-year survival for the full sample, but this negative effect appears to be stem mainly from the subset of patients unlike those in the clinical trials. Higher-staged and older patients had higher mastectomy rates and the negative effect of mastectomy on survival estimates increase with stage and age.

The Chow-test [44] *F*-values in Table 2 test whether the binary variables representing the ATR instrument in the first-stage regression of 2SLS describe a statistically significant portion of the variation in mastectomy choice. Instruments with a Chow-test *F*-statistic less than 10 are considered “weak” in the IV literature [23]. Our Chow-test *F*-statistics are much larger than 10 for both the full-sample and for all-patient subsets. Based on the Hausman statistic, the null hypothesis of whether the patient groups identified using the ATR instrument have no relationship with survival beyond the effect of the instruments on surgery choice was maintained for the full sample and all patient subsets. The 2SLS estimate of the effect of mastectomy on survival for the entire sample was negative, statistically significant, and greater in magnitude than the estimate from the linear probability model. As with the linear probability estimate, the IV estimate for patients aligned with those in the clinical trials revealed no relationship between surgery choice and survival. The negative relationship between mastectomy and survival stems mainly from patients unlike those in trials whose IV estimate is negative and statistically significant. These estimates suggest that a 1 percentage point increase in the mastectomy rate for the patients unlike those in the trial populations would reduce the 7-year survival rate by .1 percentage points. When looking at patient subsets across stage and age groups, the negative impact of mastectomy on survival appears to increase with both age and stage with statistically significant negative relationships between mastectomy use and survival for patients with stage IIa tumors and patients 81 and older. In addition, the estimates in Table 2 are robust to the number of patients used to define treatment areas around each patient ZIP code (25 or 100) and whether 6-year or 8-year survival was used as an endpoint. The estimates from these robustness checks are available from the authors.

## 4. Discussion

Substantial regional variation in mastectomy rates for patients with early stage breast cancer (ESBC) has been documented. We used the portion of mastectomy rate variation for ESBC patients 66 and older that was unrelated to measured covariates as a measure of local area mastectomy style. The survival implications associated with variation in local area mastectomy practice style for ESBC patients 66 and older were estimated in an instrumental variable (IV) analysis. In general, IV estimators produce estimates which represent the average effects of treatment on outcomes for the subset of patients whose treatment choices were influenced by the instrument specified in the analysis. Because IV estimates are specific to patients whose treatment choices are affected by the specified instrument, methodologists have cautioned about generalizing these results too broadly and making inferences using IV estimates for policy questions unrelated to the specified instrument [45]. In this paper our local area mastectomy practice style instrument is directly aligned to the policy question of whether higher mastectomy rates affect the survival of ESBC patients. 

Our IV estimates suggest that higher mastectomy rates described by differences in local area mastectomy practice styles were associated with reduced survival relative to BCSR for ESBC patients 66 and older. These estimates are consistent with the risk-adjustment estimates from linear probability models but are slightly larger. The existing NIH guideline does not mention the possibility of mastectomy survival disadvantages relative to BCSR. It suggests only that for most ESBC patients mastectomy and BCSR are equivalent with respect to survival and that mastectomy may offer advantages for higher-staged patients. However, recent evidence on radiation effectiveness from meta-analyses of controlled trials and observational studies suggests that radiation may have greater survival benefits for ESBC patients than what was presented in the original trials [46–48]. Our IV estimates support the notion that radiation provides greater survival benefits than was originally anticipated from the trial data especially for older patients with more severe disease who are unlike those patients in the original trials. In addition, it is has been shown that many BCSR patients fail to complete their adjuvant radiation therapy [49] which suggests that our results may understate the benefits of BCSR with a completed radiation course.

Of course, the inferences that can be made from our estimates depend on the validity of the assumptions underlying our estimators. Risk-adjustment estimators assume that unmeasured covariates related to surgery choice are unrelated to survival after controlling for measured covariates. It is clear from the comparison of measured covariates in Table 1 that the ESBC patients in our study that received mastectomy tended to have higher-staged disease, less well-differentiated tumors, and more comorbid conditions and were older. These relationships *suggest *that other unmeasured factors related to disease severity such as tumor location may be positively correlated with mastectomy choice. If true, our risk-adjustment estimators may be biased against mastectomy. Keating et al. showed that within plausible relationships of unmeasured confounders and surgery choice, though, that a mastectomy survival advantage cannot be supported from these data. 

Our IV estimates of the effect of mastectomy on survival are consistent if the measure of local area mastectomy practice style we used as an instrument has no direct effect on survival and is unrelated to other unmeasured factors affecting survival. As seen in Table 1, grouping ESBC patients based on local area mastectomy practice style clearly reduces the differences in measured confounders as compared to grouping patients by surgery choice. While some differences in measured covariates remained across the patients grouped by our instrument, few substantial trends remained across groups. However, it remains possible that patients grouped by local area mastectomy practice style are different in unmeasured ways that are associated with survival such as access to quality healthcare services. For example, if patients living in high mastectomy areas have less access to quality healthcare services, our IV estimates of the effect of mastectomy on survival biased be a biased low (more negative) estimated of the true effect for the ESBC patients whose surgery choices would vary with local area mastectomy practice style. Readers should also be cautioned about generalizing our IV estimates to the ESBC patients whose surgery choices would not have varied with the mastectomy practice style in the area they lived. For example, of the ESBC patients not aligned with the trial populations over 40% received mastectomy and nearly 29% received BCSR *regardless of where they lived*. This suggests there are ESBC patients that all providers believe are best-suited to a particular surgery choice regardless of the provider's practice style. Because our sample was limited to patients aged 66 and older, readers should also be cautioned about generalizing our results to younger patients.

## 5. Conclusions

The randomized controlled trial evidence that led to the guideline conclusion of survival equivalence between mastectomy and breast conserving surgery with radiation (BCSR) for patients with early-staged breast cancer (ESBC) included mainly younger, stage I patients with few comorbid conditions. As a result, a paucity of evidence is available to judge the relative effectiveness of ESBC surgery choices for patients unlike those in the trials. Observed surgery choices suggest that many providers and patients believe that mastectomy is superior to BCSR for older and higher-staged patients. However, there is no evidence to support or reject these beliefs. In this paper we were able to describe the relationship between surgery choice and survival for ESBC patients aged 66 and older by exploiting the tremendous amount of variation in local area mastectomy practice styles. Our results suggest that reducing the mastectomy rate in favor of BCSR may yield positive survival gains for higher-staged older ESBC patients. These results are probably best-applied to questions associated with changes in surgery rates across populations, and readers are cautioned about generalizing these results to all ESBC patients. Additional studies are needed to confirm these results. Future studies based on observational data should include chart abstraction components for a sample of patients to assess the validity of the assumptions underlying the estimators used. Additional controlled trials among patients unrepresented in the original ESBC controlled studies may be needed to confirm these results.

## Figures and Tables

**Table 1:** Early-stage breast cancer patient characteristics by surgery choice and instrumental variable-based patient quintile groups.

	Covariates		Surgery choice			Instrument quintile groups (higher area treatment ratio (ATR)→)					
Variable	Category	Total	BCSR	Mastectomy	*P* value (*χ* ^2^)^a^	1	2	3	4	5	*P* value (*χ* ^2^)^b^
			Column %				Column %				
Surgery	Mastectomy	16,103 (56%)	0	100%	na	37.9	50.2	59.7	65.7	68.6	<0.0001^∗^
	BCSR	12,572 (44%)	100%	0%		62.1	49.8	40.3	34.3	31.4	<0.0001^∗^

Age	66–75	16,442 (57%)	63.9	52.2	<0.0001^∗^	57.8	58.0	56.2	57.4	57.3	0.2251
	76+	12,233 (43%)	36.1	47.8		42.2	42.0	43.8	42.6	42.7	0.2251

Stage at diagnosis	Stage I	17,908 (62%)	75.1	52.6	<0.0001^∗^	63.8	62.0	62.4	60.8	63.2	0.0908
	Stage IIa	7,700 (27%)	20.1	32.1		25.7	27.5	26.7	27.8	26.6	0.1261
	Stage IIb	2,827 (10%)	4.4	14.1		9.7	9.6	10.1	10.6	9.3	0.4096
	Stage IInos	240 (1%)	0.4	1.2		0.8	0.9	0.8	0.8	0.9	0.2169

Tumor size	≤10	8,797 (31%)	40	23.4	<0.0001^∗^	31.3	30.3	30.0	29.9	31.9	0.3633
	11–20	11,958 (42%)	43.4	40.3		42.1	41.8	42.5	40.8	41.4	0.1152
	>21	7,680 (27%)	16.2	35.1		25.8	27.1	26.7	28.5	25.8	0.2090
	unknown	240 (1%)	0.4	1.2		0.8	0.9	0.8	0.8	0.9	0.2169

Grade	Well differentiated	4,751 (17%)	20.7	13.4	<0.0001^∗^	16.4	17.0	16.0	16.1	17.4	0.2426
	Moderately differentiated	10,804 (38%)	39.9	35.9		39.3	38.4	37.3	36.6	36.6	0.0002^∗^
	Poorly differentiated	6,718 (23%)	20.3	25.9		23.1	22.4	24.4	24.6	22.6	0.2138
	Undifferentiated/unknown	6,402 (22%)	19.1	24.9		21.2	22.1	22.3	22.7	23.3	0.0031^∗^

Comorbidity	Below median	15,784 (55%)	58.1	52.7	<0.0001^∗^	54.6	55.7	54.7	56.0	54.1	0.3759
	Above median	12,891 (45%)	41.9	47.3		45.4	44.3	45.3	44.0	45.9	0.3759

Physician surgical volume	Low volume	8,510 (30%)	26.0	32.6	<0.0001^∗^	27.5	33.2	30.3	29.2	28.4	0.1447
	High volume	20,165 (70%)	74.0	67.4		72.5	66.8	69.7	70.9	71.6	0.1447

Hospital bed size	≤350	15,461 (54%)	52.6	54.9	<0.0001^∗^	54.8	57.7	56.8	52.1	47.9	<0.0001^∗^
	351+	13,214 (46%)	47.4	45.1		45.2	42.3	43.2	47.9	52.1	<0.0001^∗^

Residence area size	Metro	24,530 (86%)	90.7	81.5	<0.0001^∗^	91.0	87.8	78.8	77.4	92.7	<0.0001^∗^
	Nonmetro	4,145 (14%)	9.3	18.5		9.0	12.2	21.2	22.6	7.3	<0.0001^∗^

Area median income	Above median	14,483 (51%)	53.9	47.8	<0.0001^∗^	52.4	51.9	41.8	50.2	56.3	0.0032^∗^
	Below median	13,730 (48%)	44.6	50.4		46.9	46.3	57.3	46.6	42.2	0.0001^∗^
	Missing	462 (2%)	1.5	1.7		0.7	1.8	0.9	3.2	1.5	<0.0001^∗^

^a^Test of difference in characteristic distribution between surgery choices.

^b^Cochran-Armitage test of trend in characteristic value across patients grouped into quintiles based on local area mastectomy practice style measure. For example, the *P* value for stage I tests whether a linear trend in stage I diagnoses exists across the instrument-based patient groups.

^∗^ *P* < 0.05.

**Table 2:** Estimates of the effect of mastectomy relative to breast conserving surgery with radiation on 7-year survival by estimator and sample subsets^1^.

			Estimator			
Estimation Sample	*N*	Mastectomy percentage	Linear probability estimates^2^	Instrumental variable estimates^3^		
		(1st*–*5th quintile range)	Mastectomy effect on 7-year survival (std. error)	Instrument chow test [44] *F*-value	Over identification test *F*-value	Mastectomy effect on 7-year survival (std. error)
Full sample	28,675	56.2	−0.030*	264.01*	0.60	−0.071*
		(37.9–68.6)	(0.006)			(0.029)
Trial status comparable^4^						
Yes	5,003	43.5	−0.001	50.43^∗^	0.57	0.05
		(25.5–57.0)	(0.010)			(0.05)
No	23,672	58.8	−0.037*	215.60^∗^	0.38	−0.10*
		(40.4–71.1)	(0.006)			(0.03)
Stage						
Stage I	17,908	47.3	−0.017*	174.75^∗^	0.93	−0.02
		(28.2–61.3)	(0.006)			(0.03)
Stage IIa	7,700	67.2	−0.049*	63.02^∗^	1.28	−0.15*
		(50.3–78.5)	(0.011)			(0.06)
Stage IIb	2,827	80.2	−0.085*	23.81^∗^	2.03	−0.22
		(66.4–88.7)	(0.023)			(0.13)
Age						
66–70	8,037	50.9	−0.010	75.53^∗^	1.99	0.02
		(33.5–64.3)	(0.009)			(0.05)
71–75	8,405	51.4	−0.016	81.82^∗^	0.15	−0.05
		(31.4–64.1)	(0.010)			(0.05)
76–80	6,611	57.5	−0.049*	69.94^∗^	0.42	−0.11
		(39.9–71.2)	(0.012)			(0.06)
81+	5,622	69.2	−0.065*	37.15^∗^	1.06	−0.19*
		(52.3–78.6)	(0.015)			(0.09)

^1^All models also specified all measured covariates listed in the variable definition section and are more fully described in Keating et al. [8].

^2^Average treatment effect on the treated (ATT). In this case, average effect of mastectomy on 7-year survival for those patients choosing mastectomy.

^3^Local average treatment effect (LATE). The average effect of mastectomy on 7-year survival for those patients whose mastectomy choice would have changed with local area mastectomy practice style.

^4^ESBC patients with low-risk tumors (stage I), little comorbid illness, and age ≤ 70.

*P < 0.05.
